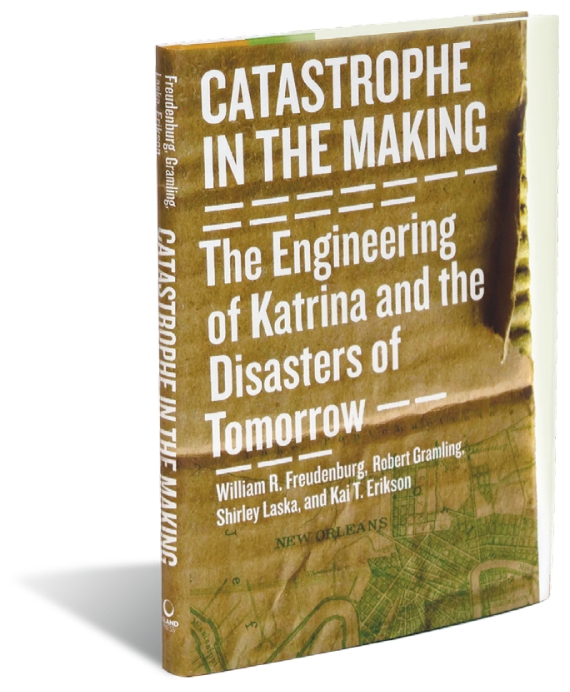# Catastrophe in the Making: The Engineering of Katrina and the Disasters of Tomorrow

**Published:** 2010-08

**Authors:** David E. Schaad

**Affiliations:** David E. Schaad, of Duke University’s Pratt School of Engineering, has designed many systems related to industrial wastewater, storm water, and flood hazards. His research focuses on sustainable engineering and development, wastewater treatment design, storm water retention/detention, and urban hydrology. As part of one of the courses he teaches, he has facilitated Spring Break experiences for more than 300 students to visit to the New Orleans area to participate in the recovery effort. Additionally, he started the DukeEngage in New Orleans program, which places approximately 10 students in volunteer internships in the region each summer

In *Catastrophe in the Making: The Engineering of Katrina and the Disasters of Tomorrow*, William R. Freudenburg, Robert Gramling, Shirley Laska, and Kai T. Erikson outline the very timely and pressing issues of moral hazards and adverse selection that unwise development practices have brought upon ourselves. Using the very tangible example of Hurricane Katrina, the authors walk readers through the colorful history of New Orleans and southern Louisiana, provide a clearly understandable description of the movement of tropical storm systems, and explain their theories on how and why Katrina had so devastating an impact on this economically, historically, and culturally important city. They rightly eschew pointing the finger of responsibility at the obvious ineptness of FEMA (the Federal Emergency Management Agency), but instead focus on the centuries-old patterns of development that weakened the Big Easy’s natural defenses against storms such as Katrina. Taking readers through the steps (and missteps) associated with how and why development occurred as it did, they draw us along on an interesting and informative history lesson in the study of a system of failures. Generally balanced in their criticism, the authors assign responsibility for the disaster to the (publicly funded) “Growth Machine” that values economic development over environmental stewardship. Their main premise is that modern-day “pirates” have directed public funds to implement (potentially) environmentally destructive projects with little societal benefit, which profit a select few and, by artificially spreading the risk and cost, do not allow the markets to act rationally.

From backgrounds primarily in sociology or environmental studies, the authors do an excellent job explaining their treatise, providing a comprehensive background on the hurricane itself—how and why it formed, the physical processes at work, and how once the storm passed the disaster started. Discussing deeper stories not widely reported in the media, they highlight the heroics of the “Cajun Flotilla” and other inventive survivors, and create a backdrop for a better understanding of the human cost associated with the storm and subsequent tragedy. They flavor all of this with an intriguing and informative overview of the historical and ethnic diversity of this area, illustrating how the people (and the environment) developed into an “ethnic and cultural mix of the region’s inhabitants [that] proved to be as rich as its famous gumbo.” Their discussion of the demise of the protective wetlands and the industrial processes that impacted the fragile cypress swamps in and around the southeastern parishes helps paint a broader picture of how development in this region could at once be so vulnerable (i.e., within the last 50 or so years building on settling soils below sea level) and so unprotected (both by degraded natural defenses and inadequately human designed and constructed systems).

All communities desire economic stability and seek to capitalize on their intellectual and human capital as well as geographic or natural resources, and at times the authors’ commentary paints (maybe rightly so) the development plans of past community leaders in a sinister light. With the hindsight of history, it is clear that some of the public works projects they attack did have unintended consequences, and the authors help establish how we as a society may “be able to *im*pair far beyond our capacity to *re*pair.” They also present an interesting economic model, and join a growing number of critics in calling for a way of quantifying and including “the full costs of a project” before it starts (or is even approved).

Freudenburg, Gramling, Laska, and Erikson correctly identify other examples of areas in the country where disasters are waiting to happen—where problems will occur because of unwise development practices. Although the authors are not engineers and hydrologists, their conclusions about how and where we develop are joining the growing cacophony of sustainable-growth proponents in calling for rational economic and development strategies to prevent this kind of tragedy from occurring again. This book provides a well-reasoned overview of the entire story of Katrina—from the historic and personal perspective to the unfolding of the problems that will continue to plague the Crescent City (and other areas with “unsafe” patterns of development).

For those unfamiliar with the story of New Orleans and the tragedy associated with Katrina, this book provides a wonderful depth and breadth that will inform and enlighten them. Those who have trod the streets of Treme, spent time in the French Quarter, haunted the streetcar as it has moved upriver on tree-lined St. Charles Avenue toward Tulane, or enjoyed the bayous in and around St. Bernard Parish will recognize the familiar story of the Mississippi River–Gulf Outlet and the personality that has swirled around the city and the different (sometimes ill-fated) economic development projects associated with the region. With regard to the conclusions the authors draw, some of their assertions regarding the hydrologic processes are questionable, but their criticisms of the development practices are solid. At times the book wears on as the authors beat the drum against the “Growth Machine,” but it is an important message that is clearly told as they encourage us to prevent the disasters of tomorrow—both in the New Orleans region and across the nation and globe where population densities are growing in hazard-prone areas. Accurately assessing the total costs (or detriments) is critically important in measuring the benefits of future projects so that as a society we can rightly attribute the value of a project and fairly appropriate the risk for the undertaking.

## Figures and Tables

**Figure:**